# High-throughput investigation of stability and Li diffusion of doped solid electrolytes via neural network potential without configurational knowledge

**DOI:** 10.1038/s41598-024-62054-7

**Published:** 2024-05-21

**Authors:** Ryohto Sawada, Kosuke Nakago, Chikashi Shinagawa, So Takamoto

**Affiliations:** Preferred Networks, Inc., Otemachi Bldg., 1-6-1 Otemachi, Chiyoda-ku, Tokyo, 100-0004 Japan

**Keywords:** Materials science, Physics

## Abstract

Solid electrolytes hold substantial promise as vital components of all-solid-state batteries. Enhancing their performance necessitates simultaneous improvements in their stability and lithium conductivity. These properties can be calculated using first-principles simulations, provided that the crystal structure of the material and the diffusion pathway through the material are known. However, solid electrolytes typically incorporate dopants to enhance their properties, necessitating the optimization of the dopant configuration for the simulations. Yet, performing such calculations via the first-principles approach is so costly that existing approaches usually rely on predetermined dopant configurations informed by existing knowledge or are limited to systems doped with only a few atoms. The proposed method enables the optimization of the dopant configuration with the support of neural network potential (NNP). Our approach entails the use of molecular dynamics to analyze the diffusion after the optimization of the dopant configuration. The application of our approach to Li$_{10}$MP$_{2}$S$_{12-x}$O$_{x}$ (M = Ge, Si, or Sn) reproduce the experimental results well. Furthermore, analysis of the lithium diffusion pathways suggests that the activation energy of diffusion undergoes a percolation transition. This study demonstrates the effectiveness of NNPs in the systematic exploration of solid electrolytes.

## Introduction

Solid-state batteries are based on solid electrolytes, which have several advantages over liquid electrolytes that make their development highly competitive. In this regard, Li$_{10}$GeP$_{2}$S$_{12}$ (LGPS) is a promising material for solid electrolytes owing to its high lithium conductivity of 12 mS cm$^{-1}$ at room temperature^1^.

Elevating the performance of solid electrolytes necessitates simultaneous improvements in their stability and Li conductivity. Atom substitution is one of the most popular approaches to improve the performance. For instance, Kato et al.^2^ studied the structure and Li ionic conductivity of Li$_{10}$(Ge$_{1-x}$M$_{x}$)P$_{2}$S$_{12}$ (M = Si, Sn) and Kuhn et al.^3^. demonstrated that Li$_{11}$Si$_{2}$PS$_{12}$ has higher ionic conductivity compared with LGPSAdditionally, recent experimental studies showed that the introduction of O as a dopant affects the stability of the LGPS phase and the activation energy of Li diffusion, which can improve the device performance. For example, O substitution in Li$_{10}$GeP$_{2}$S$_{12-x}$O$_{x}$ ($x=0.3, 0.6$) not only increases the activation energy of Li diffusion but also increases the electrochemical stability, thus enhancing the cycling performance compared with LGPS^4^. Similarly, O substitution in Li$_{10}$SiP$_{2}$S$_{12-x}$O$_{x}$ ($x<1.0$) enhances the uniformity of the LSiPSO phases and increases the Li ion conductivity in proportion to the decrease in the amount of the $\beta$-Li_3_PS_4_ phase, and growth of the LGPS-like phase^5^. However, these existing results imply that a trade-off exists between the stability and Li diffusion performance in these solid electrolytes. Therefore, it is important to elucidate the role of dopants at the atomic scale.

First-principles simulation is a promising approach for understanding these phenomena. Mo et al. investigated the phase and electrochemical stabilities of LGPS using density functional theory (DFT) simulations and found the LGPS phase to be a metastable phase that is susceptible to Li reduction^6^. The effects of the dopant were also investigated by DFT simulations. Ong et al.^7^ investigated the phase stability, electrochemical stability, and ionic conductivity of Li$_{10\pm 1}$MP$_{2}$X$_{12}$ (M = Ge, Si, Sn, Al, or P, and X = O, S, or Se) and found that decreasing the lattice parameters lowers the Li conductivity. The ionic conductivity has also been investigated using DFT simulations. For example, Yajima et al.^8^ found that the packing arrangement of Li ions governs the overall Li-ion conduction by comparing the results of nudged elastic band (NEB) calculations of the activation energy of Li diffusion with those of single-crystal neutron diffraction experiments at low-temperature. However, Li diffusion is sensitive to the atomic configuration and, apart from this, multisite hopping, in which multiple Li atoms move simultaneously, could happen^8^. Therefore, both the position of the dopant atoms and the Li diffusion path must be optimized although many previous studies have refrained from conducting these calculations because of the high computational cost.

Ab initio molecular dynamics (MD) simulation is a promising approach for determining the diffusion path of Li ions because it does not require prior knowledge of the Li diffusion pathway. Ab initio MD simulation has been applied to predict the activation energy of Li diffusion for Li$_{6}$PS$_{5}$Cl^9^, Li$_{5}$PS$_{4}$Cl$_{2}$^9^ , Li$_{10}$GeP$_{2}$S$_{12}$^10^, and Li$_{7}$La$_{3}$Zr$_{2}$O$_{12}$^10^ crystals, and to determine the grain boundaries of Li$_{7}$La$_{3}$Zr$_{2}$O$_{12}$^10^, and have successfully reproduced the experimental results qualitatively. However, ab initio MD simulations require the crystal structure to be known in advance. The required details of the crystal structure can be derived from the experimental results of the pure materials. However, it is difficult to determine the detailed configuration in the case of doped materials, and is necessary to optimize the configurations of these dopants to accurately simulate their behavior. Despite its importance, executing these optimizations through first-principles calculations is highly resource-intensive. Existing approaches usually rely on predetermined dopant configuration informed by existing knowledge or are limited to systems doped with only a few atoms^11,12^.

Neural network potential (NNP) has been developed as a tool for high-throughput calculations of physical properties. NNP emulates the energy of DFT calculations by replacing the computationally intensive electronic structure calculations with cost-effective components. Consequently, an NNP simulator that supports 72 atoms, including all the major dopant atoms for LGPS, was released^13^. This simulator enables us to investigate the role of dopants while optimizing both the dopant location and the Li diffusion pathway, in contrast to existing simulation methods.

In this study, we present a pragmatic method to screen solid electrolytes using NNP, without the need for a priori knowledge about dopant configurations or Li diffusion pathways. Our approach commences with the determination of the crystal structures by identifying the most stable arrangement among all possible dopant configurations. This facilitates the estimation of the decomposition energies without relying on prior information about the arrangement of dopant atoms. We subsequently compute the activation energy for Li diffusion within the most stable crystal structure using molecular dynamics. This method enables the identification of the optimal Li diffusion pathways without preconceived notions about them. We applied the approach to Li$_{10}$MP$_{2}$S$_{12-x}$O$_{x}$ (M = Ge, Si, Sn) and verified its agreement with previous computational and experimental results^1,3,4,7,8^. Additionally, the configuration Li$_{10}$SnP$_{2}$S$_{11.5}$O$_{0.5}$ emerged as a promising material, that is consistent with experimental result^14^. Furthermore, we propose the use of a string method to extract the minimum energy paths (MEPs) of Li diffusion from the trajectory simulated with molecular dynamics^15^. We applied the MEP analysis to O-doped LGPS to investigate the substantial increase in the activation energy observed for O-doped LMPS at specific concentrations, a phenomenon that was evident from both experimentation and simulation. Our analysis revealed that Li diffusion through O-doped LGPS avoids sites adjacent to O, thereby leading to the observed rapid rise in activation energy due to percolation transitions.

## Results and discussions

### Phase stability

**Figure 1:**
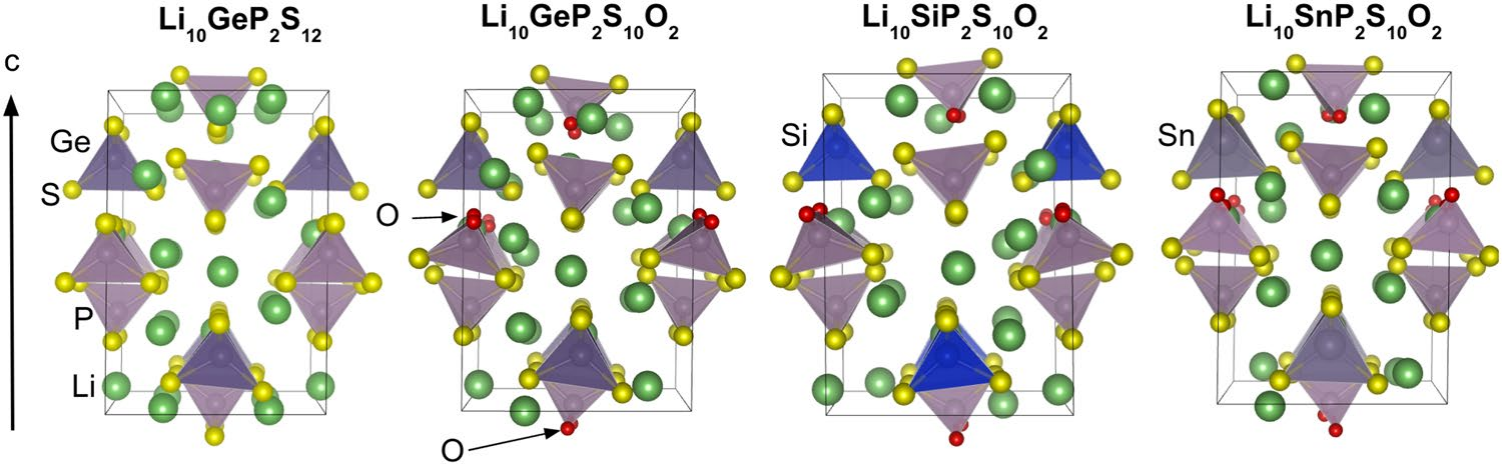
Comparison of the stable structures of LGPS, Li$_{10}$GeP$_{2}$S$_{10}$O$_{2}$, Li$_{10}$SiP$_{2}$S$_{10}$O$_{2}$ and Li$_{10}$SnP$_{2}$S$_{10}$O$_{2}$. A O atoms is substituted at S sites that construct the PS${_4}$ tetrahedra..

Table 1 presents a comparison of the lattice constants of the LMPS. The NNP calculation results are comparable to the DFT calculation results and are in good agreement with the experimental results. Figure 1 compares the stable structures of LGPS, Li$_{10}$GeP$_{2}$S$_{10}$O$_{2}$, Li$_{10}$SiP$_{2}$S$_{10}$O$_{2}$ and Li$_{10}$SnP$_{2}$S$_{10}$O$_{2}$. Evidently, one of the S atoms in the PS${_4}$ tetrahedra was always substituted by an O atom. These results are consistent with the experimental result that the largest proportion of O atoms is located at S sites that construct the PS${_4}$ tetrahedra^4^.

**Figure 2:**
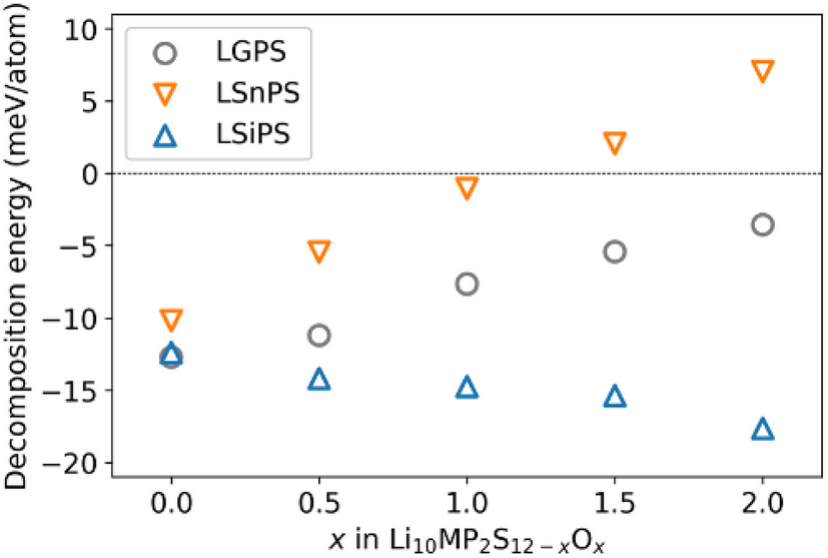
Comparison of the decomposition energy of Li$_{10}$MP$_{2}$S$_{12-x}$O$_{x}$. Higher decomposition energy implies that the Li$_{10}$MP$_{2}$S$_{12-x}$O$_{x}$ phase is more stable.

In addition, we estimated the phase stabilities of Li$_{10}$MP$_{2}$S$_{12-x}$O$_{x}$ by calculating its decomposition energy with the assumption that the decomposition products did not contain both O or S. The decomposition energy *D* is calculated as follows.

1 $$\begin{aligned} D= & {} -E_{\mathrm {Li_{10}MP_{2}S_{12-x}O_{x}}} \nonumber \\{} & {} + \left( 1-\alpha \right) \left( 2E_{\mathrm {Li_{3}PS_{4}}} + E_{\mathrm {Li_{4}MS_{4}}}\right) \nonumber \\{} & {} + \alpha \left( 2E_{\mathrm {Li_{3}PO_{4}}} + E_{\mathrm {Li_{4}MO_{4}}}\right) \end{aligned}$$

given by^6,7,16^ for M = Ge and Si and

2 $$\begin{aligned} D= & {} -E_{\mathrm {Li_{10}SnP_{2}S_{12-x}O_{x}}} \nonumber \\{} & {} + \left( 1-\alpha \right) \left( 2E_{\mathrm {Li_{3}PS_{4}}} + E_{\mathrm {Li_{4}SnS_{4}}}\right) \nonumber \\{} & {} + \alpha \left( 2E_{\mathrm {Li_{3}PO_{4}}} + \frac{1}{3} E_{\mathrm {Li_{8}SnO_{6}}} + \frac{2}{3} E_{\mathrm {Li_{2}SnO_{3}}}\right) \end{aligned}$$

for M = Sn^7^, where $E_{\textrm{material}}$ is the energy of the material and $\alpha = x/12$. A higher decomposition energy implies that the Li$_{10}$MP$_{2}$S$_{12-x}$O$_{x}$ phase is more stable. Figure 2 shows the decomposition energy of Li$_{10}$MP$_{2}$S$_{12-x}$O$_{x}$. Clearly, doping with O increases the stability of the Li$_{10}$GeP$_{2}$S$_{12-x}$O$_{x}$ and Li$_{10}$SnP$_{2}$S$_{12-x}$O$_{x}$ phases.

**Table 1:** Comparison of lattice constant of LMPS ($1 \,$Å$= 10^{-10}\,\textrm{m}$).

		a		b		c	
		[Å]	[$\%$]	[Å]	[$\%$]	[Å]	[$\%$]
LGPS	Exp.^1^	8.7177		8.7177		12.634	
	DFT^7^	8.561	− 2.8	8.847	+ 1.5	12.929	+ 2.3
	Ours	8.757	+ 0.5	8.823	+ 1.2	12.942	+ 2.4
LSiPS	Exp.^3^	8.6905		8.6905		12.5703	
	DFT^7^	8.566	− 1.4	8.848	+ 1.8	12.920	+ 2.8
	Ours	8.725	+ 0.4	8.829	+ 1.6	12.844	+ 2.2
LSnPS	Exp.^3^	8.7506		8.7506		12.799	
	DFT^7^	8.666	− 1.0	8.950	+ 2.3	13.133	+ 2.6
	Ours	8.652	− 1.1	8.894	+ 1.6	13.280	+ 3.8

### Li diffusion

**Figure 3:**
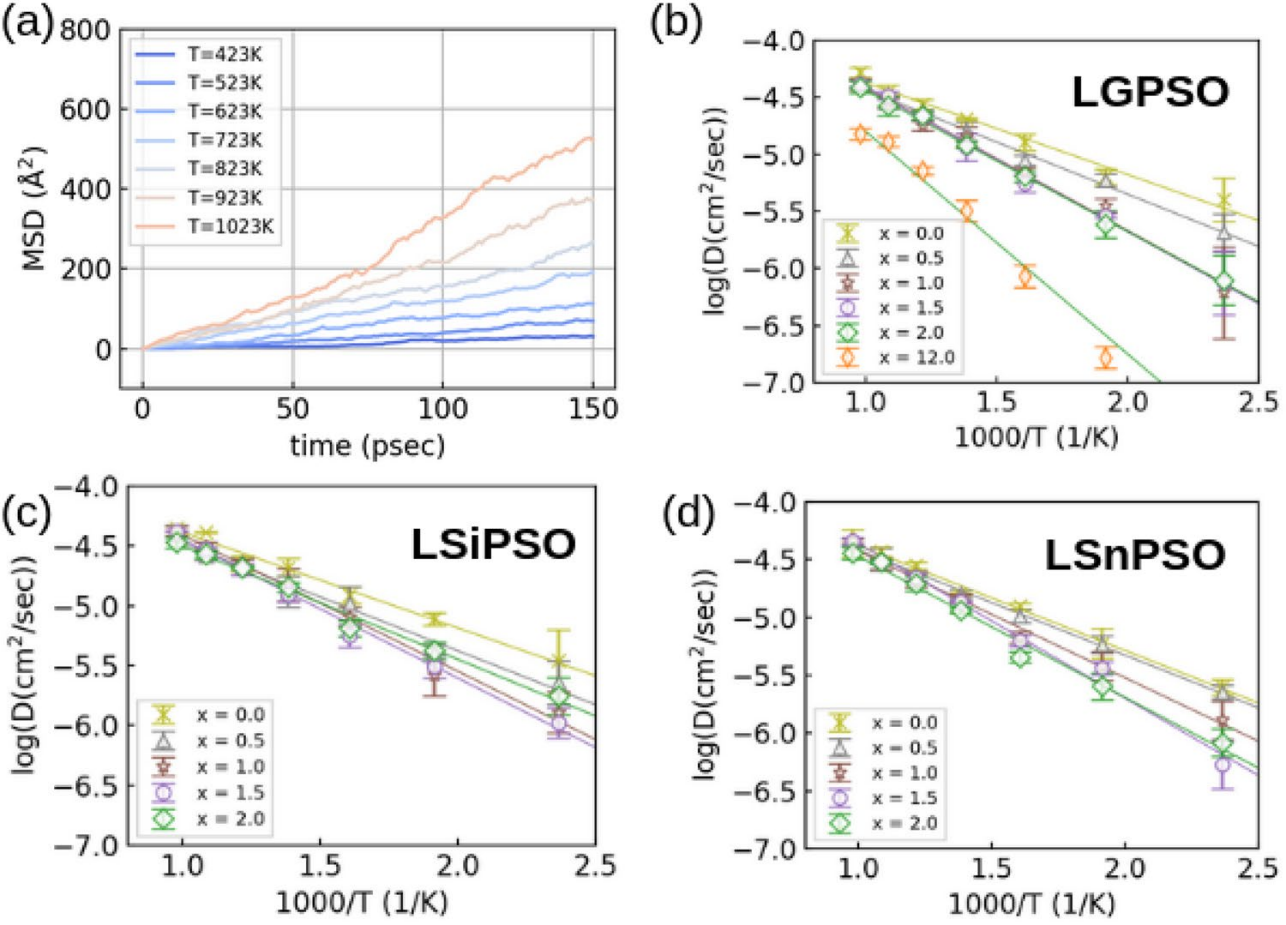
(**a**) Mean square displacement of Li. We have obtained MSD plots for all Li$_{10}$MP$_{2}$S$_{12-x}$O$_{x}$, but only the case of LGPS is shown here. (**b**)–(**d**) Arrhenius plot of diffusion coefficient for Li$_{10}$GeP$_{2}$S$_{12-x}$O$_{x}$, Li$_{10}$SiP$_{2}$S$_{12-x}$O$_{x}$, and Li$_{10}$SnP$_{2}$S$_{12-x}$O$_{x}$, respectively. The error bars correspond to statistical uncertainty in the fitting of the MSD.

**Figure 4:**
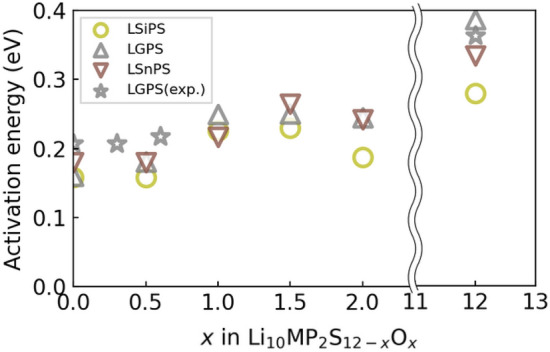
Summary of activation energy of Li$_{10}$MP$_{2}$S$_{12-x}$O$_{x}$. Experimental data are from^4^. Lower activation energy implies higher conductivity of the Li atoms.

Figure 3a shows the mean square displacements (MSD) of Li in LGPS. We have obtained these MSD plots of Li for all the Li$_{10}$MP$_{2}$S$_{12-x}O_{x}.$ By fitting the MSD plots, diffusion coefficients are calculated for each temperature. The MSD is calculated by

3 $$\begin{aligned} MSD(t)=\frac{1}{N}\sum _{i=1}^{N} <|r_{i}(t) - r_{i}(0)|^{2}> \end{aligned}$$

where *t* is the simulation time and *N* is the number of Li atoms and $r_{i}$ is the position of *i*-th Li atom, respectively^17^. Figure 3b–d show the Arrhenius plot of Li$_{10}$GeP$_{2}$S$_{12-x}$O$_{x}$, Li$_{10}$SiP$_{2}$S$_{12-x}$O$_{x}$, and Li$_{10}$SnP$_{2}$S$_{12-x}$O$_{x}$, respectively. In these figures, the activation energy corresponds to the slope of the Arrhenius plot of the diffusion coefficient. Figure 4 compares the calculated activation energies of Li$_{10}$MP$_{2}$S$_{12-x}$O$_{x}$ and the activation energy of Li$_{10}$GeP$_{2}$S$_{12-x}$O$_{x}$ obtained from the experiments^4^. The figure shows that the calculated activation energy reproduces the experimentally obtained activation energy of O-doped LGPS. Interestingly, a jump in the activation energy was observed at $x=1.0$ for all three M=Ge, Si, Sn, thus indicating a decrease in conductivity around $x=1.0$, which is consistent with experimental results^5^. However, slight ($x\sim 0.5$) O doping does not affect the activation energy, although it does contribute to the phase stability (see Fig. 2). In summary, Li$_{10}$SnP$_{2}$S$_{11.5}$O$_{0.5}$ is a promising material for solid electrolytes that can achieve both phase stability and Li mobility.

**Figure 5:**
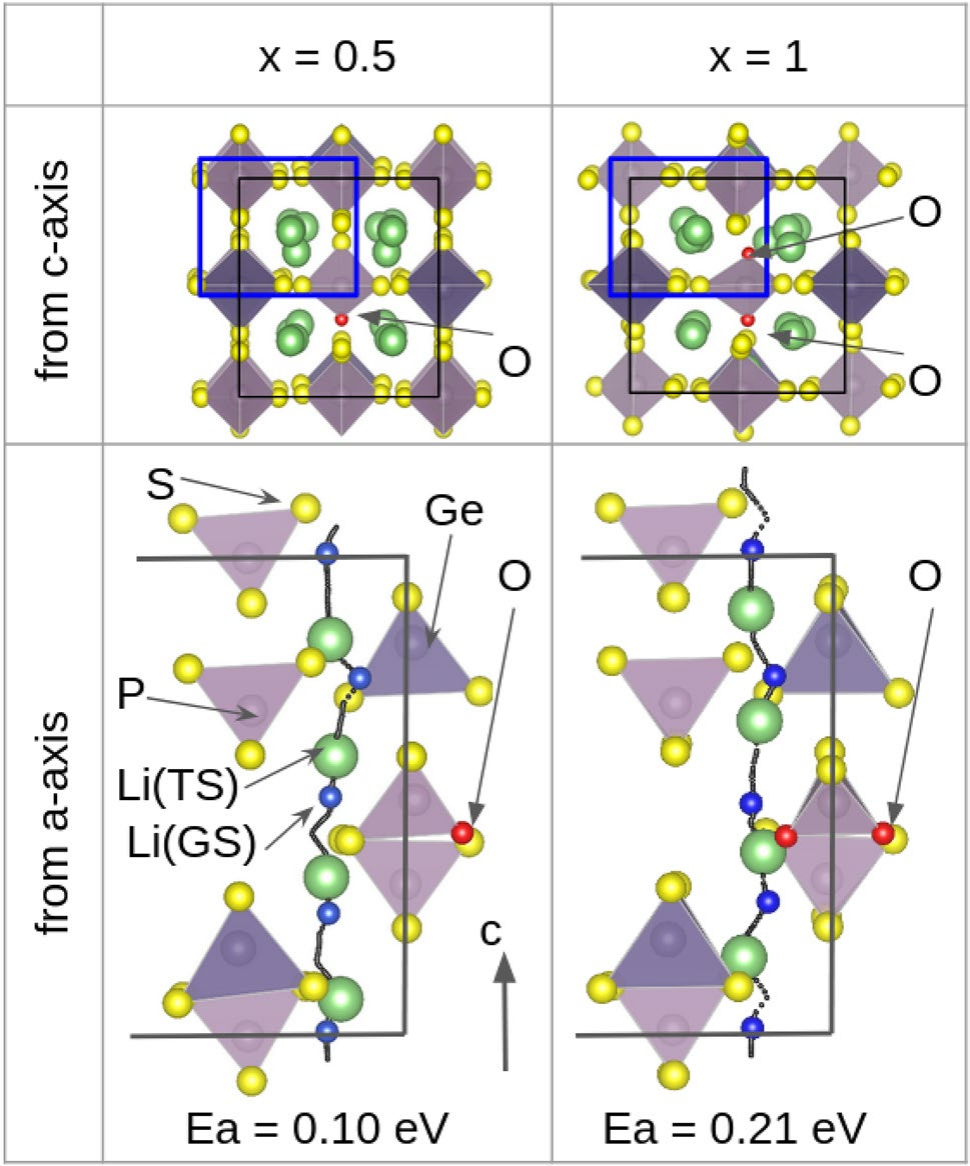
Comparison of the trajectory and activation energy ($E_{a}$) of Li diffusion via multisite hopping for an O doping concentration of Li$_{10}$GeP$_{2}$S$_{11.5}$O$_{0.5}$ ($x=0.5$) and Li$_{10}$GeP$_{2}$S$_{11.0}$O$_{1.0}$ ($x=1.0$). The black solid line indicates the boundary of the unit cell. The *a*-axis view of the trajectory shows only the atoms framed by the blue line in the *c*-axis view of the trajectory. The black dotted line in the *a*-axis view indicates the trajectories of Li atoms, where four Li atoms migrate along the *c*-axis direction simultaneously. Li atoms diffuse in the positive *c*-axis (upward) direction. The large green and small blue spheres on the trajectory indicate the positions of Li atoms in the transition and ground states, respectively.

**Figure 6:**
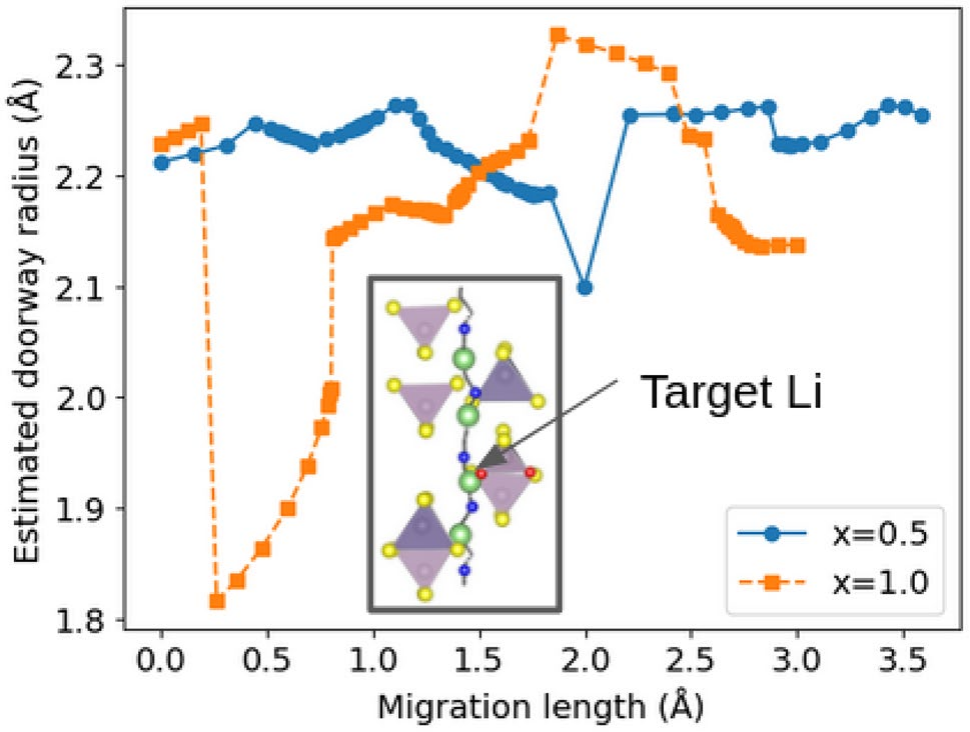
Comparison of the estimated doorway radius for the Li atom in the MEPs. The migration length is the total distance traveled by the Li atom starting from the initial state of MEP. The doorway radius of an atom is estimated by maximum radius of a sphere that can be created inside the four atoms closest to the atom. The position of the Li atom is shown in the inset.

Furthermore, we analyzed the Li diffusion path of O-doped LGPS to investigate the mechanism whereby doping causes the activation energy to increase. We extracted the Li diffusion pathways with the lowest activation energies from the respective MD trajectories of LGPS$_{12-x}$O$_{x}$ ($x=0.0, 0.5, 1.0$). The results revealed that multisite hopping, in which four of the Li atoms in the unit cell move simultaneously, has the lowest activation energy. This result is consistent with the theoretical and experimental results of Du and Yajima^8,16^, essentially suggesting that the migration of densely packed Li atoms governs diffusion. Next, we examined the extent to which the activation energy of multisite hopping changed as a result of O doping. Figure 5 compares the Li diffusion paths between the O doping concentrations of Li$_{10}$GeP$_{2}$S$_{11.5}$O$_{0.5}$ ($x=0.5$) and Li$_{10}$GeP$_{2}$S$_{11.0}$O$_{1.0}$ ($x=1.0$). The difference in the activation energies obtained from the Arrhenius plot (Fig. 4) can be attributed to a bias resulting from the fact that the calculated activation energies are for zero temperature. The plot shows the four Li diffusion channels along the *c*-axis direction from the *c*-axis view of the trajectory. For an O-doping concentration of $x=0.5$, several channels were not adjacent to the O atoms, whereas for $x=1.0$, all channels were in contact with the O atoms. Further, the positions of the O atoms were determined based on the most stable structure, as shown in Fig. 1. A comparison of the *a*-axis views of the diffusion paths shows that Li atoms are attracted to the O atoms during diffusion. Moreover, the diffusion pathway is attracted to the O atom and bends in its vicinity. These results indicate that Li atoms attracted to O atoms are less likely to move, thereby preventing multisite hopping. Additionally, we estimated the doorway radius for the Li atom in the MEPs in Fig. 6. The doorway radius becomes smaller when the Li atom passes near the O atom in MEP of $x=1.0$. This also supports the insights from the activation energies that the O atoms suppress the diffusion of Li atoms. According to our findings, the nonlinear increase in the activation energy with increasing O doping concentration, shown in Fig. 4, occurs owing to the disappearance of Li diffusion channels that are not adjacent to O atoms. Furthermore, the activation energy is expected to increase sharply at a critical O concentration.

In summary, we presented a method to screen solid electrolytes. The proposed method is based on NNP and does not require prior knowledge of the dopant configuration. Using our method, we investigated the phase stability and Li mobility of Li$_{10}$MP$_{2}$S$_{12-x}$O$_{x}$ (M = Ge, Si, or Sn) and showed this approach reproduced experimental results well. Furthermore, we analyzed the Li diffusion paths of O-doped LGPS and found that the activation energy increased with the disappearance of Li diffusion channels that are not adjacent to the O atoms. This result indicates that the percolation transition of the Li diffusion pathway occurs at a certain critical O concentration, which leads to a sharp increase in the activation energy. Estimating the specific transition point using MD calculations is challenging because it requires a huge supercell. However, it can be qualitatively predicted from the dopant configuration. For example, if a dopant other than O, such as Se, were to replace a S atom in the PS${_4}$ tetrahedron, the transition point for Se doping would be expected to be between 0.5 and 1.0. Our approach is foreseen to be applicable not only to electrolytes but also to other materials such as metal oxide and hydrogen storage materials.

## Methods

### Phase stability calculation

The crystal structure and Li occupancy of LGPS were obtained from an experimental study^4^ to calculate the stable structure of LGPS. We randomly generated 200 Li configurations that satisfy the given occupancy, optimized both the lattice constants and positions of atoms, and assumed the lowest energy structure as the stable structure. Similarly, for the O-doped LGPS, we started from the stable structure of LGPS, generated all possible dopant configurations, and repeated the abovementioned steps to obtain a stable structure. We used the L-BFGS method in an atomic simulation environment^18^ to optimize the lattice constants and positions of atoms. The same procedures were executed to obtain the structures of O-doped LSiPS and LSnPS.

### Li diffusion calculation

The activation energies of Li diffusion were calculated by MD simulations under the NVT ensemble at 423, 523, 623, 723, 823, 923, and 1023 K. The time step was chosen to be 0.5 fs, and diffusion simulations were performed for 150 ps. We used (2 $\times$ 2 $\times$ 1) supercells in the MD simulations to reduce periodic boundary effects. To decide the supercell size and the simulation time, we refered the existing study conducted in ab initio MD calculation where the supercell size was $(2 \times 1 \times 1)$ and the simulation was run at 50 ps^11^. Despite being smaller than those typically used in classical MD simulations, the trend of Li diffusion activation energy has shown good agreement with experimental results^11^. Therefore, we believe it is sufficient for the screening objectives of this study.

### MEP calculation

The MEP of the Li diffusion was taken from a molecular dynamics simulation trajectory using the following procedure. First, each Li atom in each frame of the trajectory was labeled with the site to which it belongs. Next, a Li atom was considered to have diffused when the site to which the particular Li atom belonged changed, and the frames before and after the change were used as the initial prediction of the initial and final states of Li diffusion. Finally, the reaction pathway connecting the initial state to the final state was calculated by the string method^15^. Among the multiple Li diffusion pathways calculated in this way, the one with the lowest transition state energy was adopted as the MEP.

### NNP

We used an NNP equivalent to the PreFerred Potential (PFP)^19^ version v3.0.0 on Matlantis^13^. The duration of the MD simulation for 200 atoms and 300,000 steps was approximately 10 h using an NVIDIA Tesla V100.

We verified the accuracy of NNP by comparing the atomic forces of NNP and DFT calculations.

**Figure 7:**
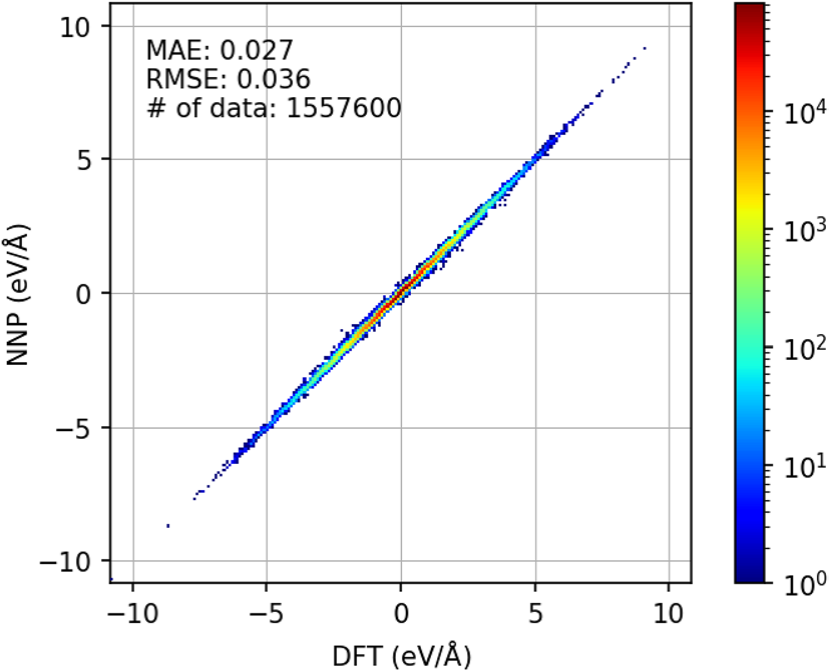
Comparison of the atomic XYZ forces of DFT and NNP for randomly extracted structures from the MD trajectories for LMPS. The color of each dot in the scatterplot indicates the number of data.

DFT calculations were performed using the the Perdew–Burke–Ernzerhof (PBE) exchange-correlation functional^20^ and the projector-augmented wave (PAW) method^21,22^ implemented in the Vienna Ab initio Simulation Package^23–26^ (VASP), version 5.4.4, with GPU acceleration^27,28^. The same calculation conditions were used to generate the PFP crystal dataset^19^. We randomly extracted 2569 structures, each containing 200 atoms, from the MD trajectories of LMPS. We compared the X-, Y-, and Z-components of the force for each atom calculated using DFT with those calculated using NNP. As shown in Fig. 7, NNP reproduces the DFT forces well over a wide range of values with a mean absolute error (MAE) of 0.027 eV/Å and root mean squared error (RMSE) of 0.036 eV/Å.

## Data Availability

The code used in the calculations and the trajectory and stable structures obtained from the calculations are included in this published article and its supplementary information files.
